# 48 Juvenile dermatomyositis: the experience of Military Pediatrics Department of Tunis

**DOI:** 10.1093/rheumatology/keac496.044

**Published:** 2022-10-07

**Authors:** Rouag Hatem, Ben Mohamed Zaineb, Dhouib Salma, Ben Rejeb Youssra, Barakizou Hager

**Affiliations:** Department of Pediatrics, Military Hospital of Tunis, Tunis, Tunisia

## Abstract

**Background:**

Juvenile dermatomyositis (JD) is a rare autoimmune disorder characterized by a non-infectious inflammatory state affecting of the muscles and skin associated with a vasculopathy which represents the essential of its physiopathogenesis.

**Objectives:**

To review the clinical and biological diagnosis of DMJ as well as its evolution under treatment based on the cases of two patients at the Military Pediatrics Department of Tunis.

**Observations:**

Case 1: This was a three-and-a-half-year-old child with a history of a simple febrile seizure, admitted for a skin rash and myalgia that had been evolving for eight months. Skin examination revealed an erythematous rash of the face and upper eyelids with erythematous papular lesions in front of the proximal interphalangeal joint recalling Gottron's papules. Muscle testing was in favor of a proximal deficiency and the electromyogram (EMG) revealed a diffuse myogenic process more pronounced on the proximal muscles without signs of myositic activity. Biology analysis did not reveal a biological inflammatory syndrome but rather increased muscle enzymes levels up to 3- and 4-times normal values. On the immune status report, there was no evidence of DMJ-specific autoantibodies (ASM), but the anti-SRP antibodies associated with myositis were present. The diagnosis of DMJ was established in view of the association of typical cutaneous signs and 3 muscular signs (proximal deficit, elevation of muscle enzymes, myogenic tracing) and an evaluation of the activity of the disease according to a standardized scale by the Childhood Myositis Assessment Scale (CMAS) was required (which was at 7/52). Treatment with corticosteroids and methotrexate was started after a pretherapeutic evaluation and regular check-ups of the child showed a clear improvement of clinical and muscular signs (CMAS became at 41/52). Case 2: A 7-year-old boy, with no previous pathological history, admitted to our department to explore his gait disorder associated to erythematosquamous lesions. At the dermatological inspection, a localized erythema was noted on the upper eyelids, the nose, the cheeks and the chin area, as well as erythematosquamous lesions on the interphalangeal joints, metacarpophalangeal joints, elbows and knees (Gottron papules). The gait was waddling, the EMG was in favor of myogenic disorder and the muscular testing revealed a proximal muscular affection. The biology results showed an elevation of CPK and LDH to 1.5 times the normal values and a sedimentation rate at 40 mm/h. The antibodies determination showed the existence of anti-NXP2 antibodies specific to myositis. The progression under treatment (corticosteroids, methotrexate and adjuvant treatment) was favorable with a CMAS score of 39 (CMAS was 11 when admitted). The persistence of squamous lesions recalling calcinosis on the dorsal surfaces of the forearms was well correlated with the presence of anti-NXP2 antibodies.

**Conclusion:**

The positive diagnosis of DMJ in a child must be based on clinical and biological arguments. The immunological characterization of DMJ should be an important element in the disease management because it can explain the lesions associated with certain myositis specific antibodies as well as their evolution, which implies a particular follow-up depending on the detected antibodies (risk of cancer in anti-NXP2 AC positive patients)

